# Efficacy and safety of lurasidone in adolescents and young adults with schizophrenia: Pooled analysis of double-blind, placebo-controlled 6-week studies

**DOI:** 10.1192/j.eurpsy.2021.442

**Published:** 2021-08-13

**Authors:** F. Calisti, I. Costamagna, J. Hsu, M. Tocco, A. Pikalov, R. Goldman

**Affiliations:** 1 Angelini Rr&d (regulatory, Research, & Development), Angelini Pharma S.p.A., Rome, Italy; 2 Medical Affairs, Sunovion Pharmaceuticals Inc., Marlborough, United States Minor Outlying Islands; 3 Medical Affairs, Sunovion Pharmaceuticals Inc., Marlborough, United States of America; 4 Medical Affairs, Sunovion Pharmaceuticals Inc, Fort Lee, United States of America

**Keywords:** schizophrénia, adolescent, lurasidone

## Abstract

**Introduction:**

Onset of schizophrenia commonly occurs during late adolescence or early adulthood and is often characterized by greater symptom severity and impairment.

**Objectives:**

To evaluate the efficacy and safety of lurasidone in the treatment of acute schizophrenia in adolescents and young adults.

**Methods:**

The 4 studies in this pooled analysis used similar study designs. Patients (ages 13-25 years) were randomized to 6 weeks of double-blind, placebo-controlled treatment with once-daily lurasidone (37 mg, 74 mg, 111 mg, 148 mg). The primary outcome was endpoint change in the Positive and Negative Syndrome Scale (PANSS) total score; secondary measures included the Clinical Global Impression, Severity scale (CGI-S).

**Results:**

The safety population consisted of 537 patients; 79.1% completed the studies. Treatment with lurasidone was significant (P<0.001) at Week 6 endpoint for change in the PANSS total score, with higher effect sizes (ES) at higher doses (37 mg, 0.53; 74 mg, 0.57; 111 mg, 0.67; 148 mg, 1.35); significance was also observed for change in the CGI-S (37 mg, 0.51; 74 mg, 0.49; 111 mg, 0.57; 148 mg, 1.75). For lurasidone (combined doses), 3 adverse events occurred with a frequency ≥5% (nausea, 13.5%; somnolence, 12.1%; akathisia, 10.1%); 4.8% of patients discontinued due to an adverse event. At LOCF-endpoint, 3.6% of patients had weight gain ≥7%, and 1.5% had weight loss ≥7%. Minimal median changes were observed at endpoint in metabolic lab values.

**Conclusions:**

In adolescents and young adults with schizophrenia, treatment with lurasidone in doses of 37-148 mg/d was a safe, well-tolerated, and effective treatment.

**Disclosure:**

Presenter is an employee of Sunovion Pharmaceuticals Inc. The study summarized in this Abstract was supported by Funding from Sunovion Pharmaceuticals Inc.

